# Mid-day photomixotrophy by *Roseiflexus* spp. and implications for the ^13^C content of hot spring cyanobacterial mats

**DOI:** 10.1128/aem.00909-25

**Published:** 2025-09-03

**Authors:** James J. Moran, Peter D. Ilhardt, Sneha P. Couvillion, Mary S. Lipton, Thomas O. Metz, Nikola Tolic, Donald A. Bryant, David M. Ward

**Affiliations:** 1 Department of Integrative Biology, Michigan State University 3078, East Lansing, Michigan, USA; 2 Department of Plant, Soil, and Microbial Science, Michigan State University 3078, East Lansing, Michigan, USA; 3 Earth and Biological Sciences Directorate, Pacific Northwest National Laboratory 6865, Richland, Washington, USA; 4 Amentum, NASA Johnson Space Center, Houston, Texas, USA; 5 Department of Biochemistry and Molecular Biology, The Pennsylvania State University 311285 , University Park, Pennsylvania, USA; 6 Department of Chemistry and Biochemistry, Montana State University, Bozeman, Montana, USA; 7 Department of Land Resources and Environmental Sciences, Montana State University, Bozeman, Montana, USA; University of Delaware, Lewes, Delaware, USA

**Keywords:** photomixotrophy, *Synechococcus*, *Roseiflexus*, stable isotope ratio, anoxygenic photosynthetic bacteria, microbial mat, hot spring

## Abstract

Microbial mats inhabiting extreme environments have been studied as modern analogs of stromatolites. Mats in Octopus Spring and Mushroom Spring, Yellowstone National Park, are predominated by unicellular photoautotrophic cyanobacteria (*Synechococcus* spp.), which are thought to cross-feed filamentous photoheterotrophic bacteria (mainly *Roseiflexus* spp.), except under early morning anoxic conditions when *Roseiflexus* spp. have been shown to fix dissolved inorganic carbon (DIC). Transcription patterns, however, suggest that *Roseiflexus* spp. may perform photomixotrophy, in which DIC is incorporated together with organic compounds during the daytime. We investigated the roles played by *Synechococcus* spp. and *Roseiflexus* spp. in DIC and organic matter uptake in mid-day light and oxic mats. Mass spectrometry was used to show that ^13^C-bicarbonate uptake under infrared (IR) light (utilized by anoxygenic phototrophs) or visible-minus-blue light (V-B) (used by cyanobacteria) was about two-thirds and one-third, respectively, of that incorporated in full light. Laser-ablation mass spectrometry analysis demonstrated that ^13^C incorporation under V-B light was restricted to the uppermost portion of the mat, whereas ^13^C incorporation under IR light was maximal in deeper mat layers. ^13^C-acetate, -propionate, -lactate, and -glycolate were incorporated to an equal or greater extent under IR and full light. Incorporation of ^13^C into peptides showed that both *Synechococcus* spp. and *Roseiflexus* spp. were active in DIC uptake, whereas *Roseiflexus* spp. exhibited greater uptake of ^13^C-organic acids, especially glycolate and lactate, into peptides. Peptides of proteins of the 3-hydroxypropionate pathway were labeled. Thus, *Roseiflexus* spp. appears to exhibit photomixotrophy throughout the day.

**IMPORTANCE:**

In these mats, *Synechococcus* spp. fix dissolved inorganic carbon (DIC) via photoautotrophy using the Calvin-Benson-Bassham cycle, which imparts a ^13^C isotopic fractionation (ε = δ^13^C_REACTANT_ - δ^13^C_PRODUCT_) of ~20‰ between DIC and photosynthate. However, the mat and DIC carbon isotope ratios suggest a much lower fractionation of ~6.4‰ to 10‰. We previously showed that *Roseiflexus* spp. fix DIC during the early morning, contributing isotopically heavier organic carbon by using the 3-hydroxypropionate pathway, which has a lower fractionation of ~13.7‰. The results of this study suggest that *Roseiflexus* spp. incorporates DIC during the day, most likely due to photomixotrophy, thus contributing to an isotopic signature heavier than expected for the Calvin-Benson-Bassham pathway. This likely also applies to other well-studied cyanobacterial mats, where it may cause the isotopic composition of mats containing cyanobacteria to approach that of mats formed exclusively by anoxygenic phototrophs, making it more difficult to distinguish these types of mats in stromatolitic organic carbon.

## INTRODUCTION

Modern microbial mats are natural extant models for the microbial communities thought to have formed stromatolites (1–3). Although stromatolites were typically formed in shallow marine settings during the Precambrian Era before the evolution of herbivorous animals, microbial mats today are limited to environments where they are protected from grazing and other disturbances. Classic examples include mats associated with lithified marine (4, 5) and freshwater (6) formations, hypersaline habitats, such as Solar Lake and Guerrero Negro (7–11), and high-temperature environments (12–16). It should be noted, however, that geothermal habitats might also have harbored early life on Earth (or other planets) (17–20).

Microbial mats in hot springs are particularly interesting, as mats formed by cyanobacteria (together with anoxygenic phototrophic bacteria) or formed exclusively by anoxygenic phototrophic bacteria are known. Molecular analyses of reaction centers (21, 22) and enzymes of chlorophyll (Chl) and bacteriochlorophyll (BChl) biosynthesis (23, 24) strongly suggest that anoxygenic phototrophy predated oxygenic phototrophy. The latter analyses further suggest that ancestral Chloroflexi-like organisms could have played an important early role in the evolution of phototrophy (23), and the ancestors of these organisms may have formed ancient stromatolites.

We studied both oxygenic and anoxygenic phototrophic systems in an attempt to discover differences that might permit them to be distinguished in the fossil record. In particular, the Calvin-Benson-Bassham pathway (CBB) used by cyanobacteria and the 3-hydroxypropionate (3-OHP) bicycle pathway used by Chloroflexi show different isotopic fractionations relative to dissolved inorganic carbon (DIC). The CBB imparts a ~20‰ fractionation (25–27), and the 3-OHP imparts an ~13.7‰ fractionation (28), and this offers the potential to distinguish oxygenic and anoxygenic mats. Interestingly, these fractionations are not reflected in bulk isotopic compositions of cyanobacterial mats and Chloroflexi mats, where isotopic fractionations are similar. For instance, isotopic compositions of Octopus Spring and Mushroom Spring cyanobacterial mat biomass relative to DIC (δ^13^C = -10.1‰ and -6.4‰) are unexpectedly similar to that for the “New Mound Annex” mat constructed by anoxygenic phototrophs (δ^13^C = -10.9‰ to -12.9‰) (29). Similarly, carbon isotopic compositions are much heavier than expected of the CBB in well-studied hypersaline mats dominated by cyanobacterial photosynthesis (30). The question arises as to why the isotopic composition of cyanobacterial mats is so heavy. Des Marais and Canfield (30) demonstrated that isotopic fractionation can become lower in systems where DIC concentrations are depleted by diffusional limitations. They also suggested that, in systems where there are multiple pathways for DIC consumption with different degrees of isotopic discrimination, the products of different community members should have different isotopic compositions.

Modeling how the inhabitants of microbial mats influence the biomarkers and isotopic signatures of the communities that might eventually become a part of the fossil record requires (i) knowledge of major native taxa, (ii) understanding the *in situ* metabolisms of taxa, especially those involved in C1 metabolism, which are most likely to introduce differences in isotopic signatures, and (iii) understanding metabolic interactions among the major taxa. Our earliest impression of the taxa inhabiting these mats was based on microscopy and cultivation of isolates thought to be representative of predominant mat inhabitants (31, 32). This led to early descriptions of the mats as being comprised of the unicellular cyanobacterium *Synechococcus lividus* and the filamentous green nonsulfur bacterium *Chloroflexus aurantiacus* (33). Molecular analyses allowed us to gain greater insight into the truly predominant mat inhabitants. For instance, 16S rRNA analyses revealed that the predominant cyanobacteria and Chloroflexi are genetically very distinct from these isolates (34). We began to refer to the predominant cyanobacteria as type A/B *Synechococcus* spp. and to the predominant Chloroflexi as type C green nonsulfur-like bacteria based on the fact that these were among the first 16S rRNA sequences we discovered (34). We later identified the type C organism as *Roseiflexus* spp., once this organism was described (35). Although we will use *Synechococcus* spp. and *Roseiflexus* spp. in the rest of the paper, those reading our earlier literature or comparing results presented in this paper to our earlier work should be aware of this evolution of the naming of mat inhabitants.

**Fig 1:**
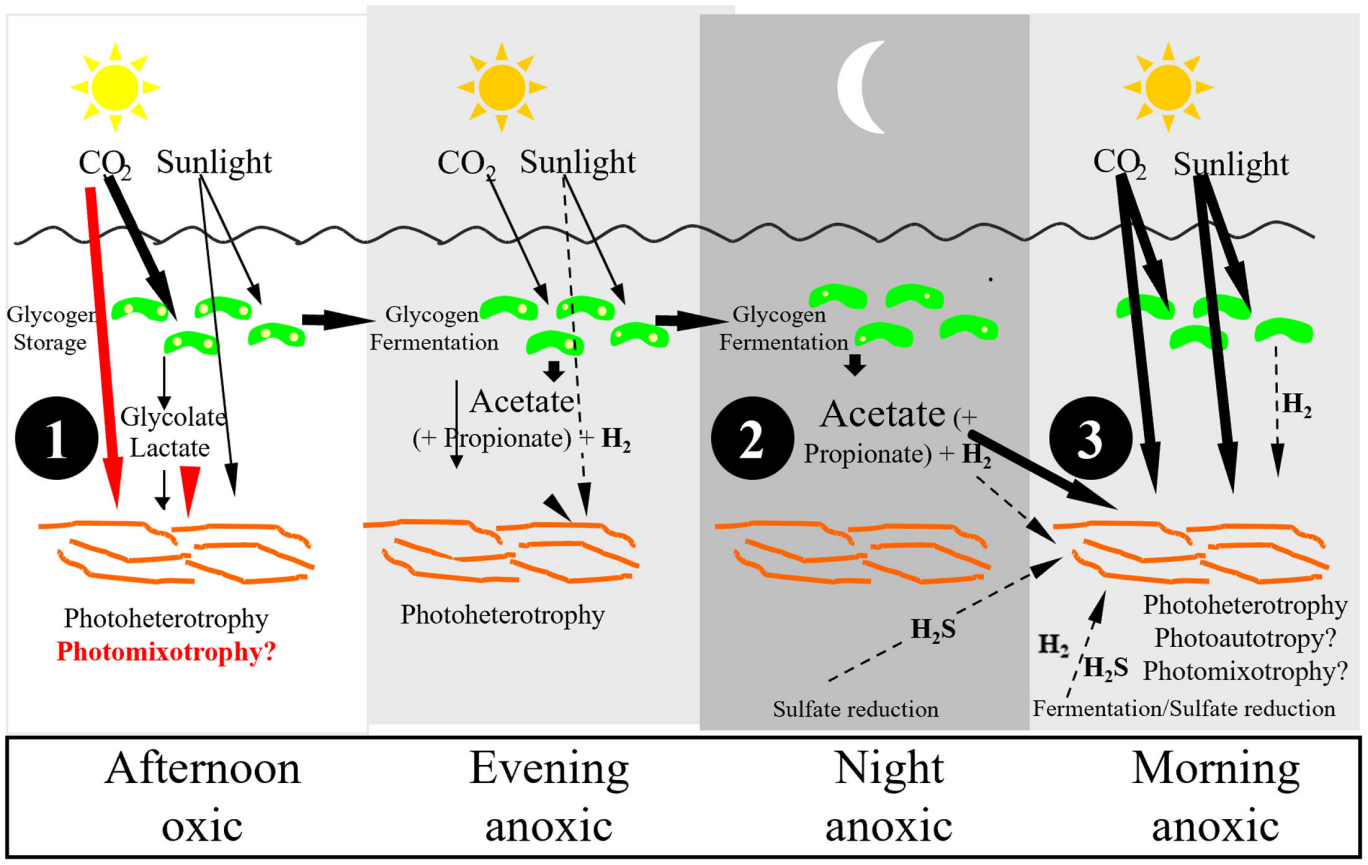
Model of carbon flow between *Synechococcus* spp. and *Roseiflexus* spp. As indicated by black arrows, it was initially hypothesized that *Synechococcus* spp. alone fixed dissolved inorganic carbon and transferred fixed carbon to support the photoheterotrophic growth of *Roseiflexus* spp. (circle 1). In the evening and through the night, the fermentation of *Synechococcus* spp. glycogen would continue to support *Roseiflexus* spp. photoheterotrophy the next morning (circle 2). During early morning, when light is too low to support enough photosynthetic oxygenic production by *Synechococcus* spp. to balance respiratory oxygen consumption and inorganic electron donors, such as H_2_ and H_2_S are present, *Roseiflexus* spp. can also fix dissolved inorganic carbon (circle 3). Red arrows show hypothesized mid-day photomixotrophy by *Roseiflexus* spp. involving simultaneous fixation of dissolved inorganic carbon and organic compounds, such as glycolate and lactate.

Assuming that *Synechococcus* spp. were the only photoautotrophs in the mat, we had initially hypothesized that these organisms supplied organic metabolites to *Roseiflexus* spp. and *Chloroflexus* spp. (black arrows and circle 1 in Fig. 1). This was based on the fact that isolates of *Chloroflexus* spp. and *Roseiflexus* spp. grew best photoheterotrophically (32, 35). Furthermore, microautoradiography was used to demonstrate uptake of ^14^C-bicarbonate by mat *Synechococcus* spp. cells and ^14^C-labeled organic metabolites by mat filamentous cells (36–38). We assumed that cross-feeding would give *Roseiflexus* spp. a carbon isotope composition similar to that of *Synechococcus* spp. However, this was not found to be the case. The *Synechococcus* spp. lipid biomarker n-heptadecane was significantly more depleted in ^13^C (−31.4‰) than *Roseiflexus* spp. wax ester biomarkers (−17.8‰ to −18.4‰) in the Octopus Spring mat (39).

We demonstrated two mechanisms by which *Roseiflexus* spp. could be enriched in ^13^C. The first involved cross-feeding from *Synechococcus* spp. Glycogen, which is the main product of bicarbonate fixation in these mats, accounting for approximately 85% of fixed carbon, is fermented in darkness to acetate (40), which, as mentioned above, mat filaments readily incorporate. Glucose in the Octopus Spring mat derived from glycogen hydrolysis was found to be isotopically heavy (−11.0‰; 29). In a pulse-labeling experiment, ^13^C-bicarbonate incorporated into the mat during the afternoon appeared to shift to *Roseiflexus* spp. wax ester biomarkers after incubation during the night, suggesting transfer of ^13^C to *Roseiflexus* spp. (41). We hypothesized that *Synechococcus* spp. glycogen fermentation products produced at night might thus be cross-fed to *Roseiflexus* spp. to support its photoheterotrophy, thus leading to isotopically heavier *Roseiflexus* spp. cell components (41, 42) (circle 2 in Fig. 1). However, after using a Percoll gradient to separate fractions of mat homogenate enriched in *Synechococcus* spp. or *Roseiflexus* spp., we observed that, surprisingly, mid-day ^13^C-bicarbonate uptake was nearly equal in these fractions (42). In this case, pulse labeling did not result in significant evidence of transfer of ^13^C in these fractions during the night.

The second mechanism involved direct incorporation of ^13^C-bicarbonate by *Roseiflexus* spp. during low-light morning periods when inorganic electron donors such as H_2_S and H_2_ are present but the mat is still anoxic because the rate of oxygen generation during oxygenic photosynthesis is lower than the rate of oxygen consumption by aerobic respiration (41–43) (circle 3 in Fig. 1). We were influenced by canonical thinking that anoxygenic photosynthesis required anoxic conditions. Thus, morning photoautotrophy (or photomixotrophy, see below) via the 3-OHP might account for why *Roseiflexus* spp. biomarkers were isotopically heavier than *Synechococcus* spp. biomarkers. Removal of infrared (IR) light decreased ^14^C-bicarbonate incorporation into the mat by 22% to 47%, suggesting that anoxygenic photosynthesis could account for a significant portion of DIC incorporation during the early morning period (41).

Metagenomic analyses provided greater insight into the metabolisms of these organisms and their pathways for fixation of inorganic carbon (44) and provided a template that permitted transcripts derived from metatranscriptomics analyses over a diel cycle to be associated with mat taxa (45, 46). Klatt et al. (47) studied transcription patterns of *Roseiflexus* spp. and, importantly, showed that transcripts encoding key enzymes of the 3-OHP pathway were expressed throughout the day. Expression patterns suggested that *Roseiflexus* spp. uses photomixotrophy (light-driven incorporation of both bicarbonate and organic metabolites) as a supply of organic carbon and reductant. Photomixotrophy by *Roseiflexus* spp. is similar to that proposed for *Chloroflexus aurantiacus* (48). Photomixotrophy was unexpected during the daytime but is consistent with the mid-day uptake of ^13^C-bicarbonate associated with *Roseiflexus* mentioned above. If *Roseiflexus* spp. perform photomixotrophy during the day, this would offer a significant explanation for why the isotopic signature of the mat is so heavy, since they fix bicarbonate using the 3-OHP (49).

Metagenomic clusters also provided the context for interpreting and understanding the mat metaproteome. We showed that the mat metaproteome closely resembles metagenomic and metatranscriptomic databases (50). The presence of key *Roseiflexus* spp. 3-OHP enzymes in mid-day metaproteomics samples provided further evidence in support of hypothesized mid-day bicarbonate fixation by this community member. Because mass spectrometry is used in proteomics analyses, we were able to develop a method similar to that used by others (51–54), which we call protein stable isotope probing (protein-SIP) to detect ^13^C incorporation into peptides of digested mat proteins. Protein-SIP enabled us to link carbon fixation with specific taxa by comparing sequences of labeled peptides to translated metagenomic cluster sequences. In the Mushroom Spring mat system, ^13^C-bicarbonate incorporation into partially labeled peptides could be recognized by shifts in mass spectra due to ^13^C incorporation in incubations ranging from 1 to 3 h. We developed an algorithm we call SIPPER for the automated detection of labeled peptides (55). We used protein-SIP and SIPPER to demonstrate the incorporation of ^13^C-bicarbonate into proteins of mat taxa during the low-light anoxic, morning time period (56). These labeled peptides were associated primarily with *Roseiflexus* (41%) and *Synechococcus* (45%). Among the labeled proteins were several peptides of key proteins of the 3-OHP pathway of *Roseiflexus*.

Here, we test the *Roseiflexus* spp. metabolism hypothesized in Fig. 1 (red arrows) of mid-day photomixotrophy based on the uptake of bicarbonate and organic compounds known to be important in the mat through metametabolomics analyses (57). We studied mid-day incorporation of ^13^C-bicarbonate under light regimes that should limit which taxa can be phototrophic. We used laser ablation mass spectral analyses to investigate the vertical distribution of ^13^C-bicarbonate incorporation. We examined the uptake of ^13^C-acetate, -propionate, -glycolate, and -lactate at times when their uptake is indicated by metametabolomics analyses. Results support the hypothesized mid-day photomixotrophy by *Roseiflexus* spp., demonstrating that the impact of the 3-OHP on ^13^C isotopic composition is much greater than previously thought and offering an explanation for why mat organic matter is isotopically heavier than if the CBB alone were affecting isotopic discrimination.

## MATERIALS AND METHODS

### Fieldwork

**Fig 2:**
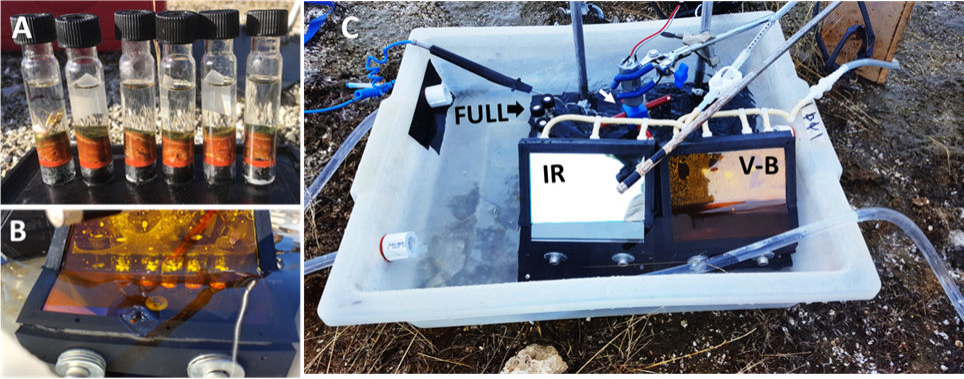
Incubation devices. (**A**) Glass screw-cap test tubes cut off at the bottom were pushed into the mat, capped, and transferred to a plastic base with O-ring seals that was fixed to a steel base plate. (**B**) A plastic “greenhouse” was placed above a set of samples and filters (here, a yellow filter to remove blue light and a hot mirror to remove infrared light) were inserted to control the light environment. White magnets were suspended in the overlying water on a fishing swivel fixed to the cap within tubes. (**C**) “Greenhouses” were incubated in a water bath of siphoned spring water. The apparatus shown in C, restricting light to visible-minus-blue (V-B), is to the right of the apparatus that uses a cold mirror to restrict light to the infrared region (IR). Samples receiving full light (FULL) were incubated outside of greenhouses. Magnets within tubes were spun using four external rotating magnets (red) powered by a motor (blue). Compressed air was introduced through latex tubing to reduce condensation.

Labeling of samples with ^13^C-bicarbonate was conducted at a ~60°C site in the microbial mat at Mushroom Spring on 4 and 5 November 2016. In advance of sampling, spring water was passed through a 0.2 µm filter, acidified to pH 4.5 (using 1 N HCl), degassed by sparging for 20 min with Ar to drive off indigenous dissolved CO_2_ species, and the pH was reset to ~8.19 (using NaOH), approximating the indigenous level. A 50 mM stock solution of ^13^C-bicarbonate was prepared by adding 20 mL of degassed water to a serum bottle containing 84 mg of ^13^C-sodium bicarbonate (Cambridge Isotope Laboratories, 99% ^13^C). Triplicate cores were taken by gently pushing sawed-off screw-cap test tubes into the mat and transferring each sample to a plastic base containing cylindrical plugs with O-rings to seal the samples beneath the core (Fig. 2A). Water above the core was removed using a long needle and syringe. This was replaced with 2.5 mL of degassed spring water, and a screw cap was then used to fix a thin butyl-rubber stopper at the top of the tube. To avoid potential unnatural thickening of the diffusive boundary layer, magnets attached to fishing spinners were suspended from beneath the cap into the water and were spun during incubation by an external rotating magnet powered by a 12 V motorcycle battery. The replicate cores were transferred to mini-greenhouse devices that were designed, constructed, and kindly provided by Biosurface Technologies Corp. (Bozeman, MT) (Fig. 2B and C). The devices were placed in a water bath kept at ~60°C by controlling the flow of hot spring effluent through the bath. Incubation was done in a shaded region of the mat during a low-light period (0933 and 1100 h with irradiance ranging from 100 to 180 uM photons/m^2^/h) and in an unshaded region during a high-light period (1350 and 1550 h with irradiance ranging from 600 to 1,000 uM photons/m^2^/h) beginning with the addition of 0.5 mL of the concentrated ^13^C-bicarbonate stock solution to achieve a final concentration of 8.33 µM, the indigenous level. Incubation was terminated by removing the samples and placing them on ice; in an attempt to allow unreacted ^13^C-bicarbonate to diffuse from the sample, the liquid was removed and replaced with 3 mL degassed spring water for 30 min before it too was removed. Tubes were then transferred to dry ice. Temperature was monitored with I-buttons. Downwelling irradiance was measured using a LiCor LI1400 light meter with a Q31191 probe.

Due to the fact that Mushroom Spring stopped flowing sometime between 6 June and 8 September 2017, labeling of samples with ^13^C-bicarbonate and ^13^C-organic compounds was conducted at a 60°C site in the microbial mat at Octopus Spring, located approximately 0.5 km from Mushroom Spring, on 18 and 19 September 2017. Molecular and chemical analyses showed that these two systems have essentially equivalent taxa and geochemistry (58, 59). Sample preparation and labeling were as described above. Stock solutions (60 mM) of sodium 1,2-^13^C-acetate, sodium 1-^13^C-propionate, 1,2-^13^C glycolic acid, and 1,2,3^13^C-L-lactic acid (all from Cambridge Isotopes, Tewksbury, MA, USA) were prepared in nondegassed Octopus Spring water and diluted upon injection into samples to achieve final concentrations of 10 mM in experimental vessels. Samples containing ^13^C-bicarbonate, ^13^C-acetate, and ^13^C-propionate were incubated for 3 h between 0930–0952 h and 1230–1255 h, and samples containing ^13^C-glycolate and ^13^C-lactate were incubated for 4 h between 1422–1525 h and 1822–1825 h, times when these compounds are decreasing in concentration in such mats (57). These samples were not stirred, and diffusional limitations likely biased label distribution toward the upper 1 mm of the mat samples (see the supplemental material). Sample collection was as above, and following incubation, samples were rinsed on ice with fresh degassed spring water to remove unreacted substrate at the time of harvest. Samples were then frozen on dry ice for transport back to the laboratory.

The light environment was controlled by using filters placed at an angle in the mini-greenhouse devices. A filter passing almost exclusively IR light (TS cold mirror (64442), Edmond Optics, Barrington, NJ; see the supplemental material) was used to largely restrict irradiance to the IR region of the light spectrum, enhancing photic metabolisms of anoxygenic mat inhabitants, which use BChls. A filter mainly blocking IR light (TS hot mirror [64462], Edmond Optics) was used in conjunction with a cutoff filter (GAM filter #425, Rosco Laboratories Inc., Stamford, CT), which blocks transmission of light between 390 and 480 nm, to restrict irradiance to mainly the visible portion of the light spectrum, minus blue light (V-B), which excites all Chls, thus providing light that could mainly excite Chl *a* used by cyanobacteria. No filter was used for samples incubated in full sunlight. The characteristics of the filters we used are presented in relation to the absorption spectra of representative *Synechococcus* and *Roseiflexus* isolates in the supplemental material.

### Bulk ^13^C measurement

Bulk isotope analyses were performed as described in Steinke et al. (56) and were used to quantify ^13^C increases in the samples with elevated levels attributable to ^13^C-bicarbonate uptake or organic substrate heterotrophic uptake. Briefly, frozen samples were sectioned across a vertical profile using a razor blade. Samples from 2016 were sectioned along three color-defined boundaries, which correlated to the dominant microbial taxa (e.g., upper, 1–2 mm thick green layer dominated by *Synechococcus* spp.; middle, 2–8 mm thick pink layer dominated by *Roseiflexus* spp.; and lower, 6–10 mm thick brown layer). The samples from 2017 were sectioned into simply upper-green and lower layers. Once sectioned, the samples were lyophilized to dryness, homogenized using a mortar and pestle, and loaded into tin capsules for analysis. We used an ECS 4010 CHNSO elemental analyzer (EA; Costech Analytical Technologies, Inc., Valencia, CA) coupled to a Thermo Scientific (Bremen, Germany) Delta V Plus isotope ratio mass spectrometer (IRMS). The samples were dropped into a combustion reactor containing cobaltic oxide and chromium oxide catalysts, maintained at 1,020°C, then the combustion products were passed through a reduction reactor loaded with copper catalyst and maintained at 650°C. In-house glutamic acid standards (δ^13^C of +16.73‰ and –11.09‰) were calibrated against U.S. Geological Survey (USGS) 40 and USGS 41 standards (60) (applied δ^13^C values of –26.39‰ and +37.63‰, respectively) and used as a basis for a two-point data correction (61). We report all bulk isotope content in standard delta (δ) notation as follows: δ = [(*R*_sample_/*R*_standard_) − 1] × 1,000, where *R*_sample_ is the measured ^13^C/^12^C ratio of a sample, and *R*_standard_ is that of an internationally recognized reference material, here Vienna Pee Dee Belemnite with an *R*_standard_ of 0.0112372.

### Laser ablation analysis

Laser ablation isotope ratio mass spectrometry was performed along a vertical profile through the samples by first manually cutting a section of the frozen sample from top to bottom (following the approach of Moran et al. [62, 63]). This section (~0.5 cm thick) was then lyophilized to dryness and placed into a laser ablation chamber in a Cetac LSX-500 system (having a 266 nm wavelength, Nd:YAG laser). Sample analysis followed the methods of Moran et al. (64). Briefly, sample areas (50 µm spot size) were manually targeted and ablated. The resulting particulates were entrained in a helium carrier gas (10 mL/min) and passed through a combustion reactor (940°C) containing platinum and nickel wires in an alumina microcombustion tube (Thermo Scientific). The resulting CO_2_ was cryotrapped in a liquid nitrogen trap, the helium flow reduced to 1 mL/min, and then the CO_2_ released into a Sercon (Cheshire, U.K.) 20-22 IRMS. All isotope ratios are reported in delta (δ) notation as used for the bulk isotope ratios, and we performed a one-point calibration of the data against an in-house standard (Premium Plus 7 monofilament 15 pound test nylon fishing line, δ^13^C = −27.71‰, as defined in Moran et al. [64]).

### Protein-SIP analysis

Proteins from incubated mat samples were extracted, trypsin-digested, fractionated by strong cation exchange chromatography, and analyzed by mass spectrometry. SIPPER was used to identify labeled peptides, using methods similar to those described in Steinke et al. (56).

#### Trypsin digestion

Protein pellets were dissolved in 200 µL of 8 M urea and mixed into solution by using a Vortex mixer. A bicinchoninic acid assay (Thermo Scientific, Waltham, MA, USA) was performed to determine protein concentration. Following the assay, dithiothreitol was added to a final concentration of 10 mM, and the samples were incubated at 60°C for 30 min with constant shaking at 800 rpm. Samples were then diluted eightfold in preparation for trypsin digestion. A total of 100 mM NH_4_HCO_3_, 1 mM CaCl_2_, and sequencing-grade modified porcine trypsin (Promega, Madison, WI, USA) were added to all protein samples at a 1:50 (wt/wt) trypsin-to-protein ratio and incubated for 3 h at 37°C with constant shaking at 450 rpm.

#### High pH fractionation

Prior to high pH reversed-phase fractionation, peptide samples were diluted to a volume of 900 µL with 10 mM ammonium formate buffer (pH 10.0), and subsequently resolved on a XBridge C18 column (250 × 4.6 mm, 5 µm) with a guard column (4.6 × 20 mm) of the same material (Waters, Milford, MA). Separations were performed at 0.5 mL/min using an Agilent 1100 series HPLC system (Agilent Technologies, Santa Clara, CA) with mobile phases (A) 10 mM ammonium formate, pH 10.0 and (B) 10 mM ammonium formate, pH 10.0/acetonitrile (10:90). The gradient was adjusted from 100% A to 95% A over the first 10 min, 95% A to 65% A over minutes 10 to 70, 65% A to 30% A over minutes 70 to 85, maintained at 30% A over minutes 85 to 95, reequilibrated with 100% A over minutes 95 to 105, and held at 100% A until minute 120. Fractions were collected every 1.25 min (96 fractions over the entire gradient). Every other row was concatenated into 24 fractions and dried completely, and 25 µL of 25 mM ammonium bicarbonate was added to each fraction for storage at −20°C until liquid chromatography tandem mass spectrometry analysis.

#### MS analysis

MS analysis of global (total protein) digest fractions was performed using a Q‐Exactive HF-X mass spectrometer (Thermo Scientific) outfitted with a homemade nano‐electrospray ionization interface. Electrospray emitters were made using a 150 µm o.d. × 20 µm i.d. chemically etched fused silica column (65). The ion transfer tube temperature and spray voltage were 300°C and 2.2 kV, respectively. Data were collected for 120 min following a 10 min delay after completion of sample trapping and start of gradient. Orbitrap spectra were acquired from 300 to 1,800 m/z at a resolution of 60 k (AGC target 3 × 10^6^) followed by data‐dependent MS/MS acquisition of the top 12 most abundant ions with an isolation window of 0.7 m/z at a resolution of 45 k (AGC target 1 × 10^5^) using a normalized collision energy of 30, dynamic exclusion time of 45 s, and detected charge state of an ion 2 to 6. The same platform was used to analyze 12 online collected fractions of total protein digest with the same arguments, except that the top 16 MS/MS spectra were acquired in data‐dependent mode with an isolation window of 0.7 m/z at a resolution of 30 k (AGC target 2 × 10^5^).

#### Data analysis

To establish a list of candidate peptides for ^13^C labeling evaluation, raw instrument spectra were first processed by MSGF+software against a proposed metagenome protein database. The database was compiled from translated and annotated metagenomes from Mushroom Spring and Octopus Spring mats updated using NCBI BLAST with the NCBI nonredundant database and a custom database representing taxocene clusters detected by Klatt et al. (44). The database, with common contaminant sequences added, contained 67,169 protein sequences. MSGF+ was used in target/decoy mode with 20 ppm parent ion tolerance, partial tryptic rule, and M oxidation (+15.9949) as dynamic modification. Best matches from the MSGF+ searches were filtered at 1% false detection rate based on the target/decoy model (66). This set of peptides was used to perform ^13^C enrichment analysis using the software SIPPER (55) with a false-positive rate of 1% (or 5% in relaxed analyses). The SIPPER-determined labeled peptides were correlated with the protein database according to the taxonomic sources and predicted functions of the proteins they represent. We determined the ^13^C-enriched peptides that appeared uniquely in one of the major taxa known to inhabit these mats (or were unique but did not fit into one of these taxa) and used the number of unique-to-taxon peptides for all quantitation except where specifically noted otherwise.

## RESULTS

### ^13^C-bicarbonate uptake into Mushroom Spring bulk mat samples in different light environments

**Fig 3:**
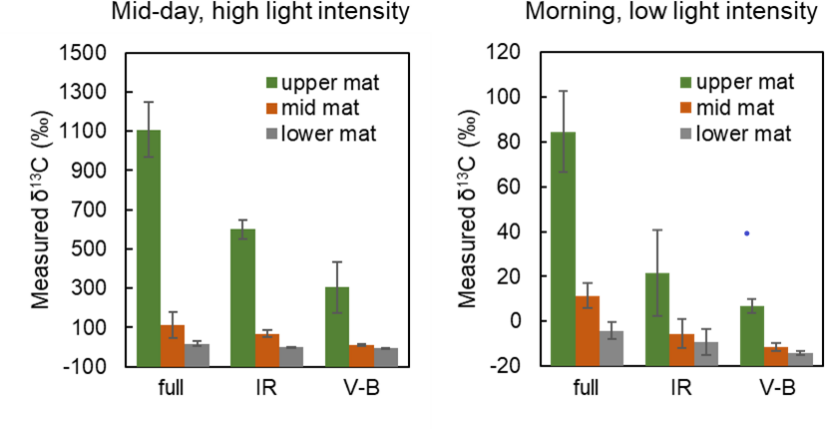
Labeling of top, middle, and bottom core segments of Mushroom Spring 60°C cores after incubation under full light, infrared light, or visible minus blue light with ^13^C bicarbonate between 0930 and 1100 h (morning) or between 1350 and 1550 h (mid-day). Error bars reflect the standard error from each set of biological incubation replicates (*n* = 3).

As shown in Fig. 3, incubation with ^13^C-bicarbonate under bright mid-day conditions (between 1350 and 1550 h) led to heavy labeling of mat cores. Most of the label was incorporated into the upper green mat layer, with significantly less label incorporation in the underlying layer and little labeling of the deeper portion of the core. Restriction to IR light significantly reduced the extent of labeling of the uppermost core section. Restriction to V-B light led to even greater reduction of labeling, and label incorporation was mainly in the uppermost layer. Comparable results were obtained for incubations done in the low-light period between 0933 and 1100 h, except that the level of label incorporation was much lower.

### Laser ablation analysis of ^13^C-bicarbonate incorporation with vertical position in the Mushroom Spring mat

**Fig 4:**
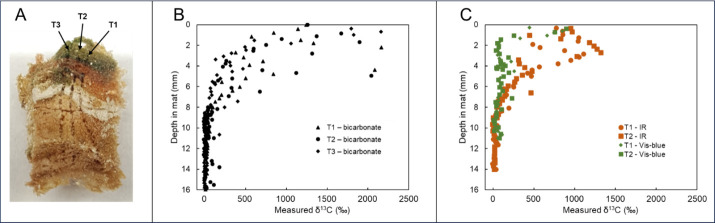
Laser ablation mass spectrometry analyses of Mushroom Spring 60°C cores labeled with ^13^C-bicarbonate between 1350 and 1550 h. (**A**) Replicate laser ablation vertical profile paths in a dehydrated core. (**B**) ^13^C enrichment at various depths of triplicate vertical paths sampled in a core incubated under full-light conditions. (**C**) ^13^C enrichment at various depths of replicate vertical profiles in cores incubated under infrared light or visible-minus-blue light.

Laser ablation mass spectrometry was used to obtain replicate vertical profiles of ^13^C bicarbonate incorporation into Mushroom Spring 60°C mat cores between 1330 and 1550 h under different light conditions (Fig. 4). For these analyses, the cores had to be dehydrated, and this caused distortion, such that distances are greater than they would be in undisturbed mat samples. To reflect this, quotation marks are used around distances in the dehydrated core. Still, these results were highly consistent with the results for labeling of bulk core samples. In full light, labeling was heaviest near the mat surface and declined with depth. Accumulation of ^13^C was measurable to approximately “10 mm” depth in the dehydrated mat slice (Fig. 4B). Restriction to IR light led to decreased labeling, mainly near the mat surface. (Fig. 4C) Although there was incorporation at the top of the core, higher levels of incorporation were observed “2–4 mm” deep in the dehydrated mat slice; again, incorporation was detected to a depth of approximately “10 mm” in the dehydrated mat slice. Restriction of light to the V-B region also resulted in less labeling than observed under full light or IR light. Labeling under V-B light was highest in the uppermost “1–2 mm” of the dehydrated mat slice, though a lower amount of labeling was observed in the subsurface at about “5 mm” depth.

### ^13^C-bicarbonate and -organic compound uptake in Octopus Spring bulk mat samples in different light environments

**Fig 5:**
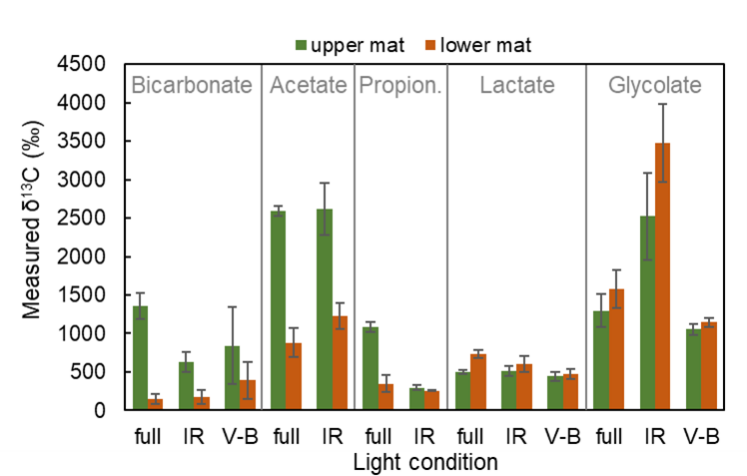
Labeling of upper and lower segments of Octopus Spring 60°C mat cores with ^13^C-labeled-bicarbonate, -acetate, or -propionate between 0930–0952 H and 1230–1255 h or ^13^C-labeled-glycolate or -lactate between 1422–1525 and 1822–1825 h under full light, infrared light, or visible minus blue light. Error bars are the standard error of biological incubation replicates (*n* = 3).

As shown in Fig. 5, ^13^C-bicarbonate was incorporated under full-light conditions between 0930–0952 and 1230–1225 h mainly in the upper green layer. Incubation under IR light and V-B light decreased the amount of incorporation. Although not statistically significant, incorporation under V-B light led to greater average incorporation than did samples incubated under IR light. However, because these samples were not stirred, diffusional limitations may have decreased the exposure of ^13^C-bicarbonate to anoxygenic phototrophs residing in subsurface layers (see the supplemental material). ^13^C-acetate was incorporated under full light during the same morning period. Most of the uptake occurred in the upper green layer, though significant incorporation was observed in the underlying brown layer. Similar results were observed under IR light conditions. ^13^C-propionate was also incorporated in full-light conditions, but incubation under IR light decreased the amount of incorporation. Less incorporation was observed in the lower mat layer. ^13^C-glycolate and -lactate were incorporated between 1422 and 1822 h, and the extent of labeling was similar in both the upper green layer and the underlying brown layer under all light conditions. ^13^C-glycolate incorporation was enhanced by incubation under IR light in both the surface and subsurface layers. Uptake under IR light conditions and labeling of both upper and lower mat regions suggest uptake of acetate, glycolate, and lactate by anoxygenic phototrophs.

### Uptake of ^13^C-bicarbonate into peptides of predominant taxa of the Mushroom Spring mat

Results of ^13^C-bicarbonate uptake by predominant taxa inhabiting the 60°C Mushroom Spring mat between 1350 and 1550 h are presented in Table 1. Replication had to be reduced to duplication due to the expense of protein-SIP analyses. Nearly half of the label incorporated into peptides under full-light conditions was found in peptides unique to *Roseiflexus* spp. Under this condition, 29.6% of the labeled peptides were associated with *Synechococcus* spp. Peptides associated with other mat community members, including other mat phototrophs (*Chloroflexus* spp., *Cand*. Thermochlorobacter spp., *Chloracidobacterium* spp., and *Cand*. Rosilinea gracilis spp.) were less heavily labeled, accounting for between 2.4% and 5.2% of the total labeled peptides and, together, comprised about 15.5% of the total number of labeled peptides. Labeling patterns were similar in samples incubated under IR and V-B light.

**TABLE 1:** Average percent labeled peptides unique to predominant taxa recovered after incorporation of ^13^C-bicarbonate into Mushroom Spring 60°C cores incubated between 1350 and 1550 h under full, infrared, and visible-minus blue light conditions^*a*^

Taxon	FULL		IR		V-B	
	Avg	SE	Avg	SE	Avg	SE
*Synechococcus*	29.6	0.2	33.4	1.0	36.8	0.1
*Roseiflexus*	48.0	0.2	44.0	1.0	40.5	0.1
*Chloroflexus*	2.4	0.0	2.5	0.1	2.4	0.0
*Thermochlorobacter*	3.0	0.8	3.4	0.1	3.6	0.5
*Chloracidobacter*	5.2	0.1	4.8	0.8	4.4	0.6
*Cand*. Roseilinea gracilis	4.9	0.6	4.7	0.3	4.2	0.0
Armatimonadetes	3.6	0.1	3.9	0.2	4.5	0.3
Klatt_Cluster_7	2.7	0.2	2.8	0.4	3.1	0.0
Klatt_Cluster_8	0.6	0.1	1.1	0.5	0.5	0.0

^*a*^ Avg, average; SE, standard error (n = 2). The total number of labeled peptides that were unique to taxa was 2,658 and 3,207 for full-light replicates, 2,746 and 3,798 for IR replicates, and 2,037 and 2,318 for visible-blue light replicates.

### Uptake of ^13^C-labeled organic acids into peptides of predominant taxa of the Mushroom Spring mat

Results from the uptake of ^13^C-labeled organic acids by peptides of predominant taxa inhabiting the Octopus Spring 60°C mat are presented in Table 2. Analysis of replicates was not possible due to the cost of protein-SIP. However, the reproducibility of triplicate bulk MS analyses of samples from which the sample we analyzed came (Fig. 3) and of duplicates in protein-SIP analyses of ^13^C-bicarbonate-labeled samples discussed in the previous section (Table 1) provides confidence in the validity of these analyses. Two patterns were observed. During morning incubations with ^13^C-acetate and ^13^C-propionate, peptides of *Roseiflexus* spp. and *Synechococcus* spp. were most heavily labeled, comprising 44.2% to 52.1% and 37.9% to 28.9% of all labeled peptides, respectively. Peptides unique to *Chloroflexus* spp., *Cand*. Thermochlorobacter spp., *Chloracidobacterium* spp., and *Cand*. Rosilinea gracilis were less heavily labeled, collectively comprising 11% to 16% of labeled peptides. During afternoon incubations with ^13^C-glycolate and ^13^C-lactate, 64% and 66.5%, respectively, of labeled peptides were unique to *Roseiflexus* spp., with *Synechococcus* spp. peptides incorporating 18.6% and 18.1% of labeled peptides, respectively. Peptides of other phototrophs accounted for 14.7% to 15.2% of the labeled peptides recovered.

**TABLE 2:** Percent of labeled peptides unique to predominant mat taxa in the Octopus Spring 60°C mat that had been incorporated during incubation under full-light conditions with ^13^C-acetate or ^13^C-propionate between 0930–0952 h and 1230–1255 h, or ^13^C-glycolate and ^13^C-lactate between 1422 and 1822 h^*a*^

Taxon	% Unique labeled peptides			
	Acetate	Propionate	Glycolate	Lactate
*Synechococcus*	37.9	28.9	18.6	18.1
*Roseiflexus*	44.2	52.1	64	66.5
*Chloroflexus*	4.1	3.2	2.8	4.9
*Cand*. Thermochlorobacter	3.3	2.9	4.4	4.9
*Chloracidobacter*	6.7	5.3	4.7	3.8
*Cand*. Rosilinea	1.5	5	3.3	1.1
*Armatimonadetes*	0.4	1.6	0.8	0.5
Klatt_Cluster_7	1.9	0.9	0.8	0
Klatt_Cluster_8	0	0.1	0.5	0

^*a*^ The total number of labeled peptides was 268, 935, 964, and 182 for acetate, propionate, glycolate, and lactate, respectively.

### Mid-day labeling of *Roseiflexus* spp. peptides of 3-hydroxypropionate bicycle pathway enzymes under full-light conditions

By relaxing the stringency of false-positive results, we were able to examine the labeling of peptides associated with *Roseiflexus* spp. enzymes involved in the 3-OHP for fixation of bicarbonate. Table 3 shows that many peptides of key enzymes of this pathway were labeled under full-light, mid-day conditions. This is consistent with the transcription results reported by Klatt et al. (47).

**TABLE 3:** Number of unique and total ^13^C-labeled peptides associated with key enzymes of the *Roseiflexus* spp. 3-hydroxypropionate bicycle detected after mid-day labeling with ^13^C-bicarbonate under full light conditions^*a*^

Enzyme	Number of unique peptides	Total peptides detected
3-Hydroxypropionyl-CoA synthetase (EC 6.2.1.36)/3-hydroxypropionyl-CoA dehydratase (EC 4.2.1.116)/acrylyl-CoA reductase (NADPH) (EC 1.3.1.84) [*Roseiflexus* sp. RS-1: NC_009523]	144	301
Acetyl-CoA carboxylase carboxyltransferase subunit alpha [*Roseiflexus* sp. RS-1: NC_009523]	20	41
Malonyl-coenzyme A reductase (NADP) (EC 1.2.1.75)/malonate semialdehyde reductase (EC 1.1.1.298) [*Roseiflexus* sp. RS-1: NC_009523]	66	136

^*a*^ The total number of labeled peptides detected that were unique to *Roseiflexus* spp. was 32,834. See Klatt et al. (47) for mid-day transcription results for these enzymes.

## DISCUSSION

Mid-day photomixotrophy by *Roseiflexus* spp. is supported by many types of evidence presented in this paper. First, light modification experiments showed that bicarbonate incorporation during the morning and early afternoon was carried out both by organisms using Chl *a* (V-B light) and by organisms using BChls (IR light). In the Mushroom Spring mat, the amount of bicarbonate incorporation under IR light was, in fact, greater than that incorporated in V-B light (Fig. 3). Second, these mats contain several anoxygenic phototrophs capable of using different wavelengths of IR light, but protein-SIP analyses (Table 1) showed that *Roseiflexus* spp. was the major organism producing labeled peptides from ^13^C-bicarbonate, with other anoxygenic phototrophs (*Chloroflexus* spp., *Cand*. Thermochlorobacter spp., *Chloracidobacter* spp., *Cand*. Roselineaea spp.) producing far fewer labeled peptides. Third, the requirement that *Roseflexus* spp. consume low-molecular weight organic compounds in photomixotrophic reactions was satisfied by the observation that acetate, propionate, lactate, and glycolate were all incorporated in IR light (Fig. 5). Again, among mat anoxygenic phototrophs, *Roseiflexus* spp. produced by far the most labeled peptides from these substrates (Table 2). Fourth, many proteins participating in the 3-OHP were labeled during mid-day (Table 3), indicating that synthesis of these proteins was occurring during high light, consistent with the observation that transcripts of the genes encoding them are expressed during the day (47). Microsensor measurements of the vertical positioning of oxygen under these conditions suggest that the mat was oxic at depths where anoxygenic photomixotrophy was occurring (67, 68).

Laser ablation analysis of the vertical distribution of ^13^C incorporated after labeling with ^13^C-bicarbonate under V-B light revealed a strong surface-associated distribution of incorporated ^13^C, with a minor peak at approximately “5 mm” depth in dehydrated samples, consistent with the pattern of distribution of *Synechococcus* species with different light adaptations (68). In contrast, incubation under IR light revealed a subsurface maximum, consistent with the distribution of the most abundant BChl-using taxon, *Roseiflexus* spp. (69), and with the strong absorption of IR light in this portion of the mat (58).

Our results are also consistent with results from microsensor measurements of vertical profiles of oxygenic photosynthesis in the Guerrero Negro saltern mat under visible or IR light (70). In that study, it was shown that removal of near-IR light caused an increase in oxygenic photosynthesis in the portion of the mat surface just beneath the peak of maximal oxygenic photosynthesis. These authors suggested that competition for DIC between anoxygenic and oxygenic phototrophs at these depths led to decreased oxygenic photosynthesis deeper in the mat and estimated that anoxygenic photosynthesis represented between 10% and 40% of DIC fixation. Oxygen profiles demonstrated that this zone was oxygenated when anoxygenic photosynthesis was taking place, and the authors pointed out that daytime anoxygenic phototrophic DIC fixation could have a significant impact on the carbon isotopic ratio of mat organic matter.

Given the lower degree of isotopic discrimination of the 3-OHP (ε = 13.7‰) compared to that of the CBB (ε = 20‰), the observation of mid-day bicarbonate fixation by *Roseiflexus* spp. has major implications for the isotopic signature of mat organic matter. Assuming that most of the light-sensitive fixation of DIC was due to the CBB and/or the 3-OHP, the impact of these two pathways can be roughly estimated by considering the relative importance of incorporation of ^13^C-bicarbonate under V-B light (i.e., by *Synechococcus* spp., the only organisms in the mat using the CBB) and under IR light (mainly by *Roseiflexus* spp., the predominant anoxygenic phototroph in the mat). As shown in Fig. 3, in the Mushroom Spring mat, about 2/3 of ^13^C-bicarbonate fixation occurs under IR light, with about 1/3 of fixation under V-B light. Similar contributions were observed in the early morning anoxic period. However, in the Octopus Spring mat (Fig. 5A), incorporation was greater under V-B light than under IR light. This could be due to the time of day at which labeling was conducted, since the Octopus Spring labeling was done in the morning (0930 to 1230 h) and Mushroom Spring mid-day labeling was performed during the afternoon. There is evidence of inhibition of oxygenic photosynthesis near the mat surface at mid-day (57, 71), thus increasing the relative importance of anoxygenic photosynthesis.

Only 2.4% to 2.5% of DIC incorporation under all light conditions was associated with peptides from *Chloroflexus* spp., which also use the 3-OHP. This is likely a result of lower population density than the two principal mat phototrophs. Similarly, proteins associated with *Cand*. Thermochlorobacter spp., *Chloracidobacter* spp., and *Cand*. Roselineaea spp., which are photoheterotrophs that do not use the 3-OHP (72), accounted for 3.0% to 3.6%, 4.4% to 5.2%, and 4.2% to 4.9% of DIC incorporation into peptides. These lower degrees of labeling could be due to lower population density, the activity of anaplerotic pathways, and/or cross-feeding from predominant photoautotrophic or photomixotrophic community members (56). These mats are complex communities, complicating the interpretation of measurements made in short-term labeling experiments.

While reduction of IR light or V-B light significantly decreased incorporation of ^13^C-bicarbonate into the mat (see Fig. 3 to 5), altered light environments did not prevent the labeling of peptides in taxa whose light-driven metabolism appears to have been reduced (see Table 1). For instance, under IR light, the number of labeled proteins detected was similar (1,067 and 1,319 in full-light replicates compared to 1,095 and 1,733 in IR-light replicates) as were the average percentages of ^13^C recovered in predominant mat taxa (Table 1). It is important to keep in mind that the number of labeled peptides represents an unknown portion of the label incorporated into the cells of a particular taxon. Nold and Ward (40) showed that shifting mat samples from light to dark conditions resulted in a shift of ^14^C-bicarbonate incorporation among the cell components of mat inhabitants. In full light, 84.6% and 9.3% of ^14^C-bicarbonate fixed into Octopus Spring mat samples was recovered in polysaccharide and protein fractions, respectively. Under dark conditions, only 5.7% of ^14^C-bicarbonate was detected in the polysaccharide fraction, with 88.4% detected in the protein fraction. Thus, although dark conditions reduced the total fixation of ^14^C-bicarbonate 13-fold, labeling of proteins was increased almost 10-fold. The restricted light environments we used were not dark, but should have been effectively dark in terms of the ability of phototrophic organisms to utilize light energy (i.e., oxygenic phototrophs “dark” under IR filter; anoxygenic phototrophs “dark” under V-B filter). Nold and Ward (40) found that under dark conditions, polysaccharides were fermented to acetate and propionate, suggesting that fermentation might drive protein synthesis when light energy is unavailable. Thus, under IR light, *Synechococcus* spp. may increase protein labeling using energy derived from fermentation, even though the total ^13^C-bicarbonate incorporated into *Synechococcus* spp. was inhibited. Under V-B conditions, the relative distribution of labeled peptides across predominant taxa was slightly shifted toward *Synechococcus* spp. Similarly, under these conditions, *Roseiflexus* spp. might have increased labeling of peptides.

Another possible explanation for peptide labeling under all light conditions is that our altered light environments did not fully exclude forms of light that could have provided energy for protein synthesis (see the supplemental material). For instance, leakage of far-red light by the cold mirror could have allowed some excitation of Chl *a*. Also, the low-light adapted *Synechococcus* spp., which reside deeper in the upper-green *Synechococcus* spp. layer of the mat, could shift their absorption into the near-IR region, allowing harvesting of light in the 700 to 800 nm range, which is present using the mostly IR-passing filter (60, 73, 74). Similarly, carotenoid pigments and bacteriorhodopsins, which are not excluded under our mostly V-B light conditions, might allow *Roseiflexus* spp. and other anoxygenic phototrophs to harvest light in the absence of IR light.

Finally, we do not know the extent to which anapleurotic pathways contribute to DIC uptake, although, as mentioned above, incorporation during dark incubation was 5.7% of that during incubation in full light (40), similar to results found in other habitats (75). Thus, these reactions are unlikely to have a significant effect on the results of light modification experiments.

Although diffusional limitations (see the supplemental material) biased uptake of organic compounds toward the upper 1–2 mm of the Octopus Spring mat, *Roseiflexus* spp. are major inhabitants of that region (69). Thus, it is clear that *Roseiflexus* spp. play a major role in the uptake of organic intermediates in the mat, likely due to their photomixotrophic metabolism. It is less clear how to explain the uptake of these compounds by *Synechococcus* spp., which we presume to be photoautotrophic. Since all of the organic compounds we studied are metabolites produced by *Synechococcus* spp., this may be due to recapture of compounds resulting from one type of metabolism (e.g., fermentation of glycogen; photorespiration) for use in another (e.g., synthesis of protein or incorporation into storage polymers). Interestingly, *Roseiflexus* spp. incorporated a higher percentage of glycolate and lactate into their proteins than did *Synechococcus* spp. This is consistent with the increase of *Roseiflexus* spp. lactate-uptake protein transcripts in the afternoon (47). Another explanation may be that oxygenic photosynthesis by *Synechococcus* spp. is less intense in the afternoon than in the morning, associated with a downward shift of the depth at which oxygenic photosynthesis occurs during the same time interval (71). This is also a time when glycolate accumulation suggests that photorespiration is occurring in *Synechococcus* spp. and lactate accumulation suggests that fermentation is occurring (58). Thus, the ability of *Synechococcus* spp. to recapture intermediates may be lower than that of *Roseiflexus* spp. at this time of day.

A major challenge in microbial community ecology is to understand the predominant community members, their *in situ* metabolisms, and interactions with other community inhabitants. In our earlier work, we had relied too heavily on readily cultivated microorganisms and metabolisms observed under laboratory conditions to interpret the results of experiments done *in situ*. For instance, we assumed that *Synechococcus* spp. were the only DIC-fixing phototrophs in the mat and that they cross-fed photosynthate to the most abundant photoheterotrophs, *Roseiflexus* spp. We also accepted the canonical thinking that anoxygenic photosynthesis required anoxic conditions to formulate an understanding of what was happening *in situ,* and this influenced us to focus investigations on *Roseiflexus* spp. DIC uptake on the early morning period, when light but anoxic conditions were present. Even when we observed that the mat percol-gradient fractions enriched in *Synechococcus* spp. and *Roseiflexus* spp. cells both incorporated significant amounts of DIC during mid-day, we were reluctant to conclude that the labeling of the *Roseiflexus* spp. fraction wasn’t due to contamination of the fraction with *Synechococcus* spp. (42). By using direct molecular approaches, we allowed nature to guide us to the true predominant taxa, their *in situ* metabolisms, and their interactions (76). Metagenomic analysis (44) provided a sequence-based means of studying *in situ* transcription and protein patterns, which led to protein-SIP methods such as those used in this study, to link activities to specific mat inhabitants. Transcription documented the *in situ* mid-day expression of 3-OHP transcripts (47), and protein-SIP produced evidence consistent with both early morning photoautotrophy (or photomixotrophy) (56) and mid-day (this study) photomixotrophy by *Roseiflexus* spp. The results of our ^13^C-DIC incorporation studies under different light regimes suggest that mid-day photomixotrophy by *Roseiflexus* spp. likely explains about two-thirds of the fixation of bicarbonate into the mat. The consequence of this activity is that the mat carbon isotope ratios are heavier than expected for the CBB-based metabolism of DIC by cyanobacterial mat inhabitants. This causes the isotopic composition of mats containing cyanobacteria to approach that of mats formed exclusively by anoxygenic phototrophs, making it more difficult to distinguish these types of mats in stromatolitic organic carbon. Diffusional limitation is likely to also affect isotopic signatures in hot spring mats, given that observed fractionations between DIC and mat organic matter are less than the fractionations expected, even if the 3-OHP were solely controlling the fractionation.

## SUPPLEMENTAL MATERIAL

Supplemental material aem.00909-25-s0001.docx Diffusional limitations in unstirred samples and absorption spectra of key organisms and filters.

ASM does not own the copyrights to Supplemental Material that may be linked to, or accessed through, an article. The authors have granted ASM a non-exclusive, world-wide license to publish the Supplemental Material files. Please contact the corresponding author directly for reuse.
