# Supplementary material for: Mid-day photomixotrophy by Roseiflexus spp. and implications for the 13C content of hot spring cyanobacterial mats
Source: Appl Environ Microbiol. 2025 Sep 3;91(10):e00909-25. doi: 10.1128/aem.00909-25 (PMC12542760; doi:10.1128/aem.00909-25)
Supplement: Supplemental material — Diffusional limitations in unstirred samples and absorption spectra of key organisms and filters. [file aem.00909-25-s0001.docx]

**Supplementary Information**

**Diffusional limitation of substrate distribution in unstirred samples.**

We decided that stirring was not necessary after comparing incorporation of ^13^C-bicarbonate in stirred (1109±10‰) and unstirred (1105±139 ‰) Mushroom Spring 60°C samples. Unfortunately, this decision was made before we conducted LA-MS analyses. Figure S1 shows an example of the vertical distribution of ^13^C-bicarbonate incorporation in an unstirred Mushroom Spring 60°C mat sample incubated in full light. Comparison to manuscript Figure 4 suggests that the distribution of added substrate was likely limited by diffusion to the uppermost “1 mm” of the dehydrated core sample. This should have biased exposure of mat taxa to the added label toward cyanobacteria, although the sample incubated under IR light shows activity within this layer. Activity in deeper layers was probably drastically reduced. A consequence of this could be the greater relative importance of incorporation of ^13^C-bicarbonate uuder V-B light than under IR light (compare Figures 3 and 5).


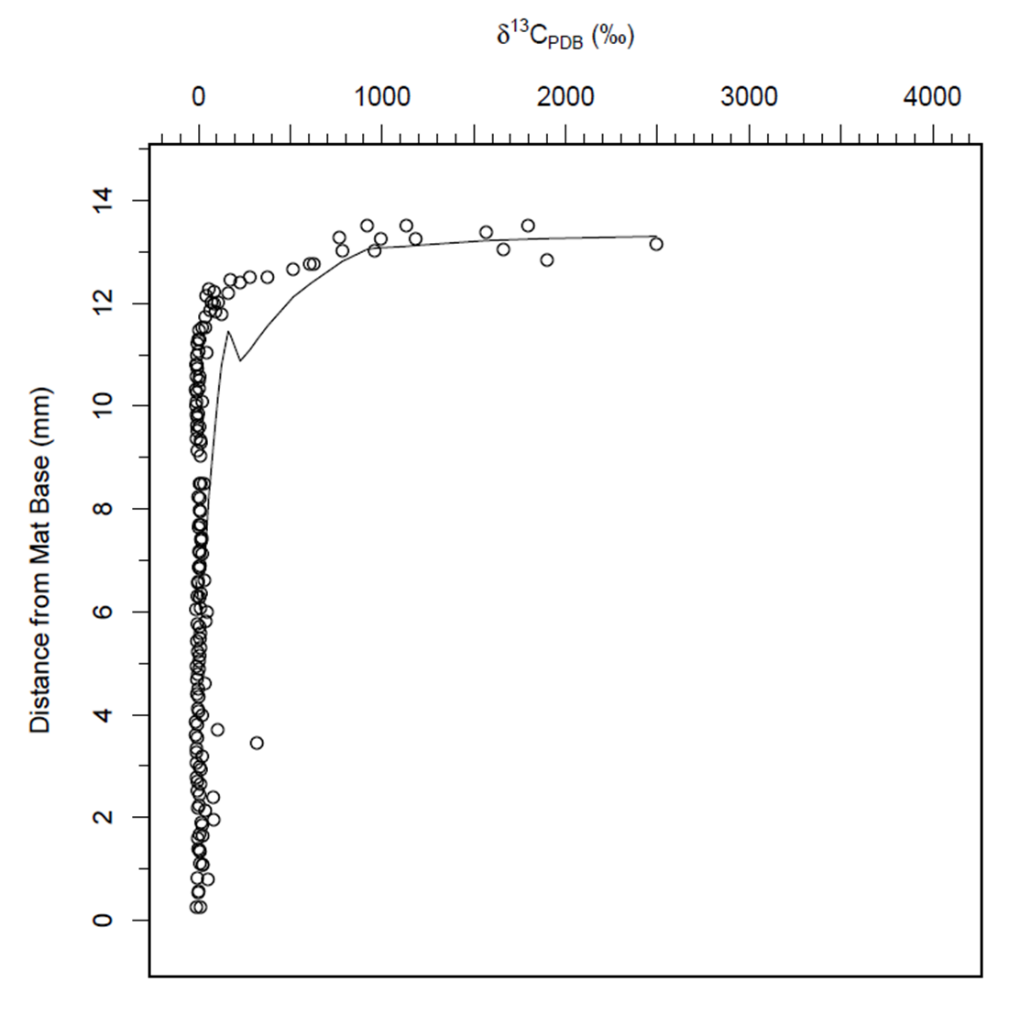


Figure S1. Laser ablation MS analysis of the vertical distribution of ^13^C-bicarbonate incorporation into a 60°C Mushroom Spring sample that was not stirred during the incubation between 1350 and 1550 h..

**Comparison of absorption spectra for representative *Synechococcus* sp. and *Roseiflexus* sp. isolates and transmission and reflectance spectra of light filters.**

As shown in Figure S2, the hot mirror transmits light at >90% between 400 and 690 nm and reflects light at >95% between 710 and 1150 nm. Between these ranges visible light is transmitted at declining intensities. The intended purpose of this mirror was to allow excitation of Chl *a* while allowing blocking activity of Bchl *b* used by *Roseiflexus* spp*.*. Since both Chl *a* and BChls are excited by blue light, a filter blocking light below 450 nm was also used (inset). The intensity of light transmitted by this filter rose to ~70% between 450 nm and 600 nm. Thus, this filter combination would allow excitement of Chl *a* at its maximum of 680 nm, but between 690 nm and 710 nm intensity would be lower. To the extent that *Roseiflexus* absorbed light between 450 and ~600 nm this might have activated *Roseiflexus*, albeit at a lower rate than without the filters.

The cold mirror reflects light at ≥95% between 400 and 690 nm and transmits light at ≥90% between 750 and 1150 nm. This would have provided IR light sufficient to excite BChl *b* of *Roseiflexus* as well as BChls of other anoxygenic phototrophs, but not the corresponding absorption maximun in the blue region of the visible spectrum. Between 690 and 750 nm IR light transmission rises. This filter could have provided some light to excite *Synechococcus* spp. pigments, especially those of low-light adapted species, whose far-red absorbance is enhanced at low light intensity (Nowack et al., 2015).

**
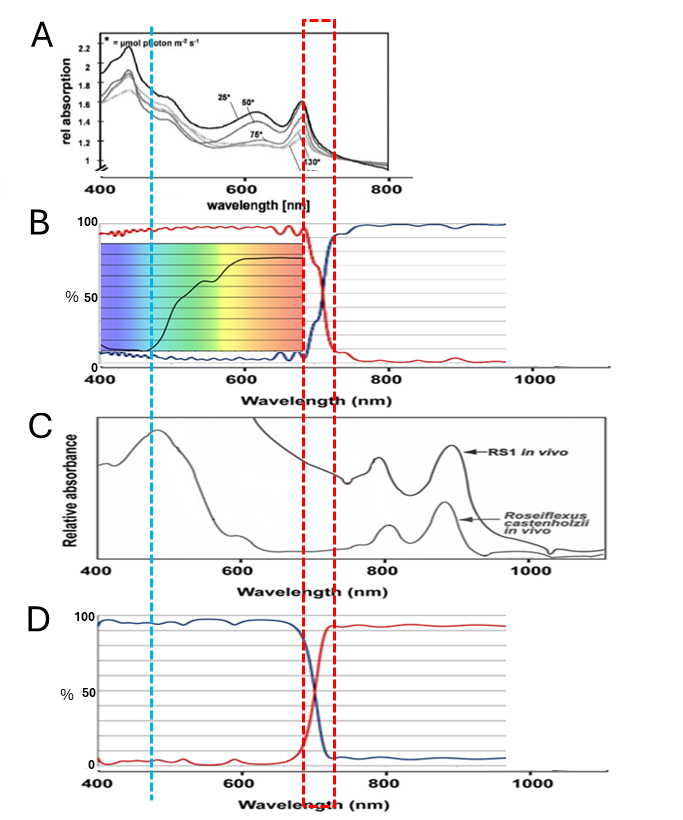
**

Figure S2. Comparison of aqueous light absorption spectra for representative *Synechococcus* sp. and *Roseiflexus* sp. isolates and reflectance/transmission spectra of cutoff filters used to predominately excite the photopigments of one organism or the other. A. Absorption spectra for *Synechococcus* spp. strain CIW-10, derived from strain B' cultivated from the Octopus Spring mat (Allewalt et al., 2006) measured at different light intensities (from Kilian et al., 2007). B. Transmittance (red line) and reflectance (blue line) for hot mirror filter. Inset shows transmission spectrum for filter used to exclude blue light, which is slightly raised relative to the vertical scale to enable viewing of both spectrat. C. Absorption spectra of two *Roseiflexus* isolates, one of which, strain RS1, was cultivated from the Octopus Spring mat; the other strain was cultivated from a Japanese hot spring (Hanada et al., 2002). D. Transmission (red line) and absorption (blue line) of cold mirror. A is from Killian et al. (2007); B and E are from the Edmonds Optics website; insert in B is from the Rosco Laboratories website https://legacy.rosco.com/mycolor/SEDpdf.cfm?titleName=G425:%20Sunflower&imageName=../images/filters/gam/G425.pdf); C is from van der Meer et al. (2010).

**References**

Allewalt, J.P., M.M. Bateson, N.P. Revsbech, K. Slack, and D.M. Ward. 2006. Effect of temperature and light on growth of and photosynthesis by *Synechococcus* isolates typical of those predominating in the Octopus Spring microbial mat community of Yellowstone Naational Park. Appl. Environ. Microbiol. 72:544-550.

Hanada, S., S. Takaichi, K. Matsuura, K. Nakamura. 2002. *Roseiflexus castenholzii* gen. nov., sp. nov., a thermophilic, flamentous, photosynthetic bacterium that lacks chlorosomes. Intl. J. System. Evol. Microbiol. 52:187-193.

Kilian, O., A.-S. Stenou, F. Fazeli, S. Bailey, D. Bhaya, and A.R. Grossman. 2007. Responses of a thermophilic *Synechococcus* isolaate from the microbial mat of Octopus Spring to light. Appl. Environ. Microbiol. 73:4268-4278.

Nowack S., M.T. Olsen, G. Schaible, E.D. Becraft, G. Shen,I. Klapper, D.A. Bryant and D.M. Ward. 2015. The molecular dimension of microbial species: 2. *Synechococcus* isolates representative of putative ecotypes inhabiting different depths in the Mushroom Spring microbial mat exhibit different adaptive and acclimative responses to light. Frontiers in Microbiology 6: 626.
